# Associations of *BAFF* rs2893321 polymorphisms with myasthenia gravis susceptibility

**DOI:** 10.1186/s12881-019-0906-8

**Published:** 2019-10-30

**Authors:** Hui Deng, Jianjian Wang, Xiaotong Kong, Huixue Zhang, Tianfeng Wang, Wenqi Tian, Tingting Yi, Lihua Wang

**Affiliations:** 1 Department of Neurology, The Second Affiliated Hospital of Harbin Medical University, 246 Xuefu Road, Harbin, 150081 Heilongjiang China; 2 Department of Neurology, The Affiliated Hospital of Inner Mongolia Medical University, Huhehaote, 010050 Inner Mongolia China

**Keywords:** *BAFF*, rs2893321, Polymorphisms, Myasthenia gravis

## Abstract

**Background:**

Myasthenia gravis (MG) is an autoimmune diseases characterized by fatigue and weakness of skeletal muscles. B-lymphocyte-activating factor (*BAFF*), an essential factor for B cell differentiation and development, is important in the progression of MG. The current study aimed to investigate the association between single nucleotide polymorphism rs2893321 in *BAFF* with MG susceptibility in Chinese Han population.

**Methods:**

One hundred forty-nine patients with MG and 148 healthy controls were recruited. Using improved multiple ligase detection reaction technology, the polymorphisms of rs2893321 between groups and among MG subgroups have been compared.

**Results:**

A significant differences between the MG group and the healthy control group was observed. Additionally, rs2893321 was found to be associated with gender and age in patients with MG.

**Conclusion:**

Genetic variations of rs2893321 in *BAFF* might be associated with susceptibility to MG in the Chinese Han population.

## Background

Myasthenia gravis (MG) is an autoimmune neuromuscular disorder mediated by pathogenic autoantibodies. MG is characterized by fatigue and weakness of skeletal muscles [1]. It is relatively rare, with a prevalence rate of 40 to 80 per million and an annual incidence rate of 4 to 12 per million [2]. In about 80% of patients with MG, acetylcholine receptor (AChR) is the autoantigen, and antibodies against AChR can be detected [3]. In AChR-negative patients, antibodies against other neuromuscular junction proteins, such as low-density lipoprotein receptor-related protein 4 (LRP4) and muscle specific kinase (MuSK), are usually observed [4]. Although the pathogenesis in MG has been elucidated, the underlying mechanism of this disease remains unclear.

MG is a B-cell-mediated disease [5]. B cells maintain normal adaptive immune responses by regulating T cell function, inflammatory cytokine production, and antibody formation [6]. B-lymphocyte-activating factor (*BAFF*), which belongs to the tumor necrosis factor family, is an essential factor for B cell differentiation and development [7]. *BAFF*-stimulated B cell proliferation and maturation prolongs survival by binding to the *BAFF* receptor in B cell membranes [8]. In animal models, *BAFF* decline can lead to B cell deficiency, whereas *BAFF* overexpression facilitates B cell proliferation and elevates serum antibody levels [9]. Evidence shows that dysregulation of *BAFF* contributes to autoimmune disorders, including rheumatoid arthritis, systemic lupus erythematosus, and primary biliary cirrhosis [10, 11]. Serum *BAFF* level also are elevated in MG. Additionally, *BAFF* positively regulated anti-AChR antibody in MG patients, which indicates *BAFF* may play an important role in the pathogenesis of MG [12, 13].

Although the precise genetic origin of MG is unclear, the polymorphisms of several candidate genes have been implicated [14]. Specifically, *BAFF* polymorphisms are associated with the phenotypes and occurrence of autoimmune diseases [15]. The variant, single nucleotide polymorphism (SNP) rs2893321 of the *BAFF* gene, has been reported to be a susceptible genetic variant for the development of Graves’ disease and autoimmune thyroid diseases [9]. Thus, we hypothesized a connection between rs2893321 and the occurrence and clinical characters of MG. In the current study, we examined the polymorphisms of *BAFF* rs2893321 in Chinese patients with MG and in healthy controls to determine its association with genetic susceptibility to MG.

## Methods

### Study population

From October 2015 to April 2017, we enrolled 149 patients with MG and 148 healthy controls from the Second Affiliated Hospital of Harbin Medical University. All participants were unrelated members of the Han Chinese population. Patients in the MG group met the standard MG diagnosis: fluctuating muscle weakness; positive neostigmine test; and an amplitude decrease of low-frequency, repetitive nerve stimulation exceeding 10%. Most patients with MG also were AChR antibody positive, as detected via radioimmunoprecipitation assay. And all patients were undergo CT exam to text for thymoma. The healthy controls had no diagnosed neurological or autoimmune diseases. Basic information (age and gender) of patients and controls were recorded. The ethical committees of the Second Affiliated Hospital of Harbin Medical University approved this study. All participants provided informed consent.

### Sample collection

Peripheral venous blood samples were collected from all participants and stored in blood-collecting vessels contained ethylenediamine tetra-acetic acid (EDTA). Genomic DNA was isolated from each blood sample using a QIAamp Blood Midi Kit (Qiagen, Beijing, China). Concentrations of all DNA samples were measured by nucleic acid spectrophotometer. The A_260_/A_280_ of DNA samples from 1.8 to 2.0 were selected and stored at − 20 °C.

### Single nucleotide polymorphism genotyping

Genotyping of *BAFF* rs2893321 was performed using improved multiple-ligase detection reaction (iMLDR) technology (Shanghai Genesky Biotechnologies Inc., Shanghai, China), which is based on multiplex fluorescence PCR. To ensure double-blinded quality control, we selected 4% samples from the MG and healthy control groups, respectively. The results were consistent with the original SNP genotyping data.

### Statistical analysis

All data were analyzed by SPSS 22.0 software. Data are presented as mean ± SD and analyzed by *t* test. The χ^2^ and Fisher exact texts were used to compare genotype and allele frequencies between the MG group and healthy control group and MG subgroups (sex, age, with or without thymoma, etc.). *P* values < .05 were considered significant. SNPstats (http://bioinfo.iconcologia.net/snpstats/start.htm) was used to detect the Hardy-Weinberg equilibrium in healthy controls. Logistic regression was used to adjust for potential confounders affecting differences between the MG group and heathy controls and among MG subgroups.

## Results

### Characteristics of subjects in the myasthenia gravis and control groups

We enrolled 149 patients with MG (68 women and 81 men) aged 49.35 ± 15.17 years and 148 healthy controls (72 women and 76 men) aged 49.58 ± 14.92 years. Although the female gender ratio was slightly higher in the MG group than in the control group, there were no significant differences in age or gender (*P* > .05). Among patients with MG, 56 were diagnosed with ocular MG and 93 with general MG; 70 had thymoma, and 79 had no thymoma; and 51 were AChR antibody positive, and 98 were AChR antibody negative.

### *BAFF* rs2893321 polymorphisms and myasthenia gravis

As shown in Table 1, we observed significant differences in genotype frequencies of *BAFF* rs2893321 between the MG group and the control group (χ^2^ = 6.088, *P* = .048). Frequency of genotype GG in MG patients (18.1%) was significantly elevated compared to control group (10.8%) (*P* < 0.05). We observed no significant difference in allele frequency of rs2893321 between MG patients and healthy control subjects.

**Table 1:** Genotype and allele frequencies of *BAFF* rs2893321 in patients with myasthenia gravis and in healthy controls

	Control *n* (%)	MG *n* (%)	χ^2^ value	*P* value
Genotype				
AA	52 (35.1%)	61 (40.9%)	6.088	.048*
GA	80 (54.1%)	61 (40.9%)		
GG	16 (10.8%)	27 (18.1%)		
Allele				
A	184 (62.2%)	183 (61.4%)	0.036	.850
G	112 (37.8%)	115 (38.6%)		

* Myasthenia gravis group versus control group, *P* < .05

### *BAFF* rs2893321 and gender in patients with myasthenia gravis and in healthy controls

As shown in Table 2, rs2893321 genotype frequencies were significantly different between women in the MG group and those in the control group (χ^2^ = 6.145, *P* = .046). Frequency of genotype AA in female MG patients (48.1%) was significantly elevated compared to control group (31.6%) (*P* < 0.05).

**Table 2:** Genotype and allele frequencies of *BAFF* rs2893321 in male or female subjects

	Male				Female			
	Control n (%)	MG n (%)	χ^2^	*P*	Control n (%)	MG n (%)	χ^2^	*P*
Genotype								
AA	28 (38.9%)	22 (32.4%)	3.821	.148	24 (31.6%)	39 (48.1%)	6.145	.046*
GA	36 (50.0%)	30 (44.1%)			44 (57.9%)	31 (38.3%)		
GG	8 (11.1%)	16 (23.5%)			8 (10.5%)	11 (13.6%)		
Allele								
A	92 (63.9%)	74 (54.4%)	2.603	.107	92 (60.5%)	109 (67.3%)	1.555	.212
G	52 (36.1%)	62 (45.6%)			60 (39.5%)	53 (32.7%)		

*MG group versus control group, *P* < 0.05

### *BAFF* rs2893321 and age in patients with myasthenia gravis patients and in healthy controls

Participants were divided into two groups: younger than 50 years (EOMG) and older than 50 (LOMG). As shown in Table 3, the rs2893321 genotype frequencies in the EOMG group showed significant differences between the MG and control groups (χ^2^ = 6.058, *P* = .048). Frequency of genotype AA in EOMG patients (43.0%) was significantly elevated compared to control group (34.2%) (*P* < 0.05).

**Table 3:** Genotype and allele frequencies of *BAFF* rs2893321 in patients younger and older younger than 50 years

	< 50 years old				> 50 years old			
	Control n (%)	MG n (%)	χ^2^	*P*	Control n (%)	MG n (%)	χ^2^	*P*
Genotype								
AA	26 (34.2%)	34 (43.0%)	6.058	0.048*	26 (36.1%)	27 (38.6%)	.979	.613
GA	42 (55.3%)	29 (36.7%)			38 (52.8%)	32 (45.7%)		
GG	8 (10.5%)	16 (20.3%)			8 (11.1%)	11 (15.7%)		
Allele								
A	94 (61.8%)	97 (61.4%)	0.007	0.935	90 (62.5%)	86 (61.4%)	.035	.852
G	58 (38.2%)	61 (38.6%)			54 (37.5%)	54 (38.6%)		

*MG group versus control group, *P* < .05

### *BAFF* rs2893321 polymorphisms among myasthenia gravis subgroups

The MG group was divided into patients with and without thymoma. As shown in Table 4, no significant differences in genotype and alleles of rs2893321 were observed. Additionally, in all 149 patients with MG, no significant differences in rs2893321 polymorphisms were observed between patients diagnosed with ocular MG and general MG.

**Table 4:** Genotype and allele frequencies of *BAFF* rs2893321 in MG subgroups

	Myasthenia gravis n (%)				Myasthenia gravis n (%)			
	Thymoma	Non-thymoma	χ^2^	*P*	Ocular	General	χ^2^	*P*
Genotype								
AA	30 (42.9%)	31 (39.2%)	1.796	0.407	20 (35.7%)	41 (44.1%)	1.883	.390
GA	25 (35.7%)	36 (45.6%)			23 (41.1%)	38 (40.9%)		
GG	15 (21.4%)	12 (15.2%)			13 (23.2%)	14 (15.1%)		
Allele								
A	85 (60.7%)	98 (62.0%)	0.054	0.817	63 (56.2%)	120 (64.5%)	2.016	.156
G	55 (39.3%)	60 (38.0%)			49 (43.8%)	66 (35.5%)		

### *BAFF* rs2893321 polymorphisms in AChR antibody positive and AChR antibody negative patients with myasthenia gravis

As shown in Table 5, we observed no significant difference of rs2893321 polymorphisms between patients with MG who were AChR antibody positive and negative.

**Table 5:** Genotype and allele frequencies of *BAFF* rs2893321 in AChR antibody positive and AChR antibody negative patients with myasthenia gravis

	AChR antibody status		χ^2^ value	*P* value
	Positive	Negative		
Genotype				
AA	18 (35.3%)	43 (43.9%)	1.856	.395
GA	21 (41.2%)	40 (40.8%)		
GG	12 (23.5%)	15 (15.3%)		
Allele				
A	57 (55.9%)	126 (64.3%)	1.999	.157
G	45 (44.1%)	70 (35.7%)		

## Discussion

In the current study, the single nucleotide polymorphism rs2893321 of the *BAFF* gene was found to be associated with susceptibility to MG in a Chinese Han population. Several clinical variables can affect analysis of gene polymorphisms. Thus, we adjusted the MG clinical features to determine whether the polymorphisms of rs2893321 were independent risk factors for a specific variable. Significant differences were found between the rs2893321 genotype and women with MG and MG patients younger than 50 years. No associations were found between the rs2893321 polymorphism and MG patients with or without thymoma or MG patients who were AChR antibody negative or positive.

*BAFF* stimulates the biological functions of B cells, and dysregulation of *BAFF* can affect the development of MG [16]. The genetic variant of *BAFF* is associated with systemic lupus erythematosus, Graves’ disease, chronic lymphocytic leukemia, and other autoimmune diseases [17–19]. However, no study has investigated the connection between *BAFF* polymorphisms and MG. In our study, the results demonstrated that the frequencies of the AA genotype of rs2893321 were significantly higher in patients with MG than in the healthy controls, which suggests that rs2893321 might be associated with MG susceptibility in this ethnic Chinese Han population. However, our study population size was relatively small compared to other case-control studies. To confirm our result and to discover more detail of rs2893321 in MG, additional study with larger samples are needed.

In this study, rs2893321 was found to be closely associated with MG patients who were younger than 50 years and female. On the other hand, no significant differences in allele were observed among MG patients in different age and gender. We suspect it is because the frequency of GA genotype was high and had no significant different between groups, and then cause the allele A and G showed no significant changes. Additionally, no significant differences in genotype or allele frequency of rs2893321 were observed among MG patients with or without thymoma or MG patients who were AChR antibody positive and negative. These results suggest that the genetic polymorphism of rs2893321 might be restricted to specific subgroups of MG.

It has been reported that MG can be divide into different age subgroups, early-onset MG (EOMG, younger than 50 years) and late-onset MG (LOMG, older than 50) [20]. The management strategies and treatment can be different due to the age difference [21]. Our study indicated the genotype frequency of rs2893321 was different in EOMG patients compared with healthy control, which suggested rs2893321 in *BAFF* might be a susceptible genetic variant in EOMG patients. Thus people with rs2893321 variant should pay more attention to prevent the development of MG in a relatively early age.

After logistic regression analysis, the results indicated that the genetic impact of rs2893321 was gender-dependent. In brief, the genetic effect of the rs2893321 AA genotype appeared to be more significant among women than among men. This finding may be attributable to differences in basic immune responses due to different sex hormones [22]. Gender indeed affects the association between genetic polymorphisms and susceptibility to MG [23]. It has been reported that *BAFF* gene expression can affect sex hormones in animals and in humans. A study has shown *BAFF* to be negatively correlated with testosterone and positively correlated with estrogen [24]. These results suggest that sex hormones affected *BAFF* gene expression and that the genetic variant of rs2893321 in *BAFF* was significantly associated with susceptibility to MG in women.

## Conclusions

This study demonstrate the association between *BAFF* polymorphisms and MG. In a Chinese Han population, rs2893321 was found to be associated with MG susceptibility, specifically in women with the condition and in patients who were younger than 50 years. Future studies should expand the patient population and investigate more SNPs in the *BAFF* gene to understand the connection between *BAFF* and MG.

## Data Availability

The datasets used and/or analysed during the current study available from the corresponding author on reasonable request.

## Abbreviations

AChR: Acetylcholine receptor
*BAFF*: B-lymphocyte-activating factor
EDTA: Ethylenediamine tetra-acetic acid
EOMG: Early-onset myasthenia gravis
iMLDR: Improved multiple-ligase detection reaction
LOMG: Late-onset myasthenia gravis
LRP4: Low-density lipoprotein receptor-related protein 4
MG: Myasthenia gravis
MuSK: Muscle specific kinase

## References

[1] Evoli A (2017). Myasthenia gravis: new developments in research and treatment. Curr Opin Neurol.

[2] Dalakas MC. Immunotherapy in myasthenia gravis in the era of biologics. Nat Rev Neurol. 2018;15(2):113–124. 10.1038/s41582-018-0110-z 30573759

[3] Carvalho TP, Vianna CL, Andrade FF, Alvarenga RA, Sales LOM, Rodrigues RS, Rosado-de-Castro PH (2018). Asymmetric pattern in generalized myasthenia gravis: a case report. Medicine (Baltimore).

[4] Ben Younes T, Benrhouma H, Klaa H, Ben Aoun R, Rouissi A, Ben Ahmed M, Kraoua I, Ben Youssef-Turki I. Muscle-specific kinase autoimmune myasthenia gravis: report of a pediatric case and literature review. Neuropediatrics. 2018;50(2):116–121. 10.1055/s-0038-1676514 30577044

[5] Stathopoulos P, Kumar A, Heiden JAV, Pascual-Goni E, Nowak RJ, O'Connor KC (2018). Mechanisms underlying B cell immune dysregulation and autoantibody production in MuSK myasthenia gravis. Ann N Y Acad Sci.

[6] Koulouri V, Koutsilieris M, Mavragani CP (2019). B cells and atherosclerosis in systemic lupus erythematosus. Expert Rev Clin Immunol.

[7] Smulski CR, Eibel H (2018). BAFF and BAFF-receptor in B cell selection and survival. Front Immunol.

[8] Xu H, He X, Xu R (2018). B cell activating factor, renal allograft antibody-mediated rejection, and long-term outcome. J Immunol Res.

[9] Lin JD, Yang SF, Wang YH, Fang WF, Lin YC, Lin YF, Tang KT, Wu MY, Cheng CW (2016). Analysis of associations of human BAFF gene polymorphisms with autoimmune thyroid diseases. PLoS One.

[10] Wei F, Chang Y, Wei W (2015). The role of BAFF in the progression of rheumatoid arthritis. Cytokine.

[11] Feng GD, Xue XC, Gao ML, Wang XF, Shu Z, Mu N, Gao Y, Wang ZL, Hao Q, Li WN (2014). Therapeutic effects of PADRE-BAFF autovaccine on rat adjuvant arthritis. Biomed Res Int.

[12] Kang SY, Kang CH, Lee KH (2016). B-cell-activating factor is elevated in serum of patients with myasthenia gravis. Muscle Nerve.

[13] Ibtehaj N, Huda R (2017). High-dose BAFF receptor specific mAb-siRNA conjugate generates Fas-expressing B cells in lymph nodes and high-affinity serum autoantibody in a myasthenia mouse model. Clin Immunol.

[14] Jiang P, Yue YX, Hong Y, Xie Y, Gao X, Gu CK, Hao HJ, Qin Y, Ding XJ, Song M (2018). IL-4Ralpha polymorphism is associated with myasthenia gravis in Chinese Han population. Front Neurol.

[15] Nezos A, Papageorgiou A, Fragoulis G, Ioakeimidis D, Koutsilieris M, Tzioufas AG, Moutsopoulos HM, Voulgarelis M, Mavragani CP (2014). B-cell activating factor genetic variants in lymphomagenesis associated with primary Sjogren’s syndrome. J Autoimmun.

[16] Avidan N, Le Panse R, Harbo HF, Bernasconi P, Poulas K, Ginzburg E, Cavalcante P, Colleoni L, Baggi F, Antozzi C (2014). VAV1 and BAFF, via NFkappaB pathway, are genetic risk factors for myasthenia gravis. Ann Clin Transl Neurol.

[17] Marin-Rosales M, Cruz A, Salazar-Camarena DC, Santillan-Lopez E, Espinoza-Garcia N, Munoz-Valle JF, Ramirez-Duenas MG, Oregon-Romero E, Orozco-Barocio G, Palafox-Sanchez CA (2019). High BAFF expression associated with active disease in systemic lupus erythematosus and relationship with rs9514828C>T polymorphism in TNFSF13B gene. Clin Exp Med.

[18] Lane LC, Allinson KR, Campbell K, Bhatnagar I, Ingoe L, Razvi S, Cheetham T, Cordell HJ, Pearce SH, Mitchell AL (2019). Analysis of BAFF gene polymorphisms in UK Graves’ disease patients. Clin Endocrinol.

[19] Jasek M, Bojarska-Junak A, Wagner M, Sobczynski M, Wolowiec D, Rolinski J, Karabon L, Kusnierczyk P (2016). Association of variants in BAFF (rs9514828 and rs1041569) and BAFF-R (rs61756766) genes with the risk of chronic lymphocytic leukemia. Tumour Biol.

[20] Fan L, Ma S, Yang Y, Yan Z, Li J, Li Z (2019). Clinical differences of early and late-onset myasthenia gravis in 985 patients. Neurol Res.

[21] Gilhus NE, Tzartos S, Evoli A, Palace J, Burns TM, Verschuuren J (2019). Myasthenia gravis. Nat Rev Dis Primers.

[22] Pakpoor J, Goldacre R, Goldacre MJ (2016). Low testosterone and myasthenia gravis in males: a national record-linkage study. J Neurol.

[23] Gregersen PK, Kosoy R, Lee AT, Lamb J, Sussman J, McKee D, Simpfendorfer KR, Pirskanen-Matell R, Piehl F, Pan-Hammarstrom Q (2012). Risk for myasthenia gravis maps to a (151) Pro-->Ala change in TNIP1 and to human leukocyte antigen-B*08. Ann Neurol.

[24] Panchanathan R, Choubey D (2013). Murine BAFF expression is up-regulated by estrogen and interferons: implications for sex bias in the development of autoimmunity. Mol Immunol.

